# Women’s views about physical activity as a treatment for vasomotor menopausal symptoms: a qualitative study

**DOI:** 10.1186/s12905-020-01063-w

**Published:** 2020-09-14

**Authors:** Adèle Thomas, Amanda J. Daley

**Affiliations:** 1 Office of HDR Training and Partnerships, Macquarie University, Sydney, NSW Australia; 2 School of Sport, Exercise and Health Sciences, Loughborough University, Loughborough, Leicestershire LE11 3TU UK

**Keywords:** Exercise, General practice, Hot flushes, Menopause, Night sweats, Primary care

## Abstract

**Background:**

Women commonly seek medical advice about menopausal symptoms. Although menopausal hormone therapy is the most effective treatment, many women prefer non-pharmacological treatments, such as physical activity. The effectiveness of physical activity has been inconclusive when assessed by randomised controlled trials, and it remains unclear how women feel about it as a possible treatment approach. The aim of the study was to explore symptomatic menopausal women’s views and experiences of physical activity as a treatment for vasomotor and other menopausal symptoms.

**Methods:**

An in-depth qualitative study was embedded within a randomised controlled trial that assessed the effectiveness of physical activity as a treatment for vasomotor menopausal symptoms in previously inactive vasomotor symptomatic women. Participants were randomised to one of two physical activity interventions or a usual care group. Both physical activity interventions involved two one-to-one consultations, plus either supporting materials or access to physical activity support groups, over 6 months. Semi-structured interviews were conducted with 17 purposively selected participants from all three trial groups after they had completed trial follow-up. Interviews were audio recorded, transcribed verbatim, and analysed by constant comparison.

**Results:**

All participants talked positively about physical activity as a treatment for their menopausal symptoms, with most reporting participation had improved their hot flushes and night sweats. They reported that they had experienced improved sleep, physical health and psychological well-being. Those who received the physical activity plus social-support intervention reported their ability to cope with their menopausal symptoms had improved. Many participants commented that they would prefer doctors to discuss physical activity as a possible treatment for their hot flushes and night sweats, before offering medication.

**Conclusions:**

Based on the views and experiences of the women who participated in this study, healthcare professionals should continue discussing physical activity as a potential first treatment option with menopausal women. Furthermore, healthcare professionals should ensure they prepare, support, and encourage these women both physically and emotionally.

**Trial registration:**

ISRCTN ISRCTN06495625 Registered 10/11/2010

## Background

Menopause is a natural phase of a woman’s life that typically occurs when they are around 47–50 years [1]. For many women this period can be a difficult physiological and psychological transition with around 45% of women seeking professional advice for their menopausal symptoms [2–6]. The symptoms experienced include vasomotor (hot flushes and night sweats: HF/NS), psychological (e.g. depression and, insomnia), somatic/physical (e.g. palpitations, backache and, dizziness), and sexual (reduced libido and, vaginal dryness). The principal symptom usually responsible for women seeking professional help is HF/NS [2–4], which can persist for many years [7].

The most effective treatment for HF/NS is menopausal hormone therapy (MHT). Nonetheless, there has been a decline in the use of MHT [8, 9] due to concerns about potential associated adverse risks [10], and women are keen to try non-pharmacological treatments. One such treatment is physical activity. Several trials have investigated the effectiveness of physical activity as a treatment for vasomotor and other menopausal symptoms, but findings have been inconsistent. A recent systematic review concluded that there was insufficient evidence on whether physical activity is an effective treatment for HF/NS [11].

Despite the uncertainty about the effectiveness of physical activity as a treatment option for HF/NS, it is well evidenced that physical activity has many benefits for physical and psychological health [12–14]. Of relevance to menopausal women, are the benefits of regular physical activity for reducing the risk of cardiovascular disease, osteoporosis, obesity, and depression. It is important therefore to continue to explore whether physical activity can reduce HF/NS and whether physical activity is an acceptable treatment option for the management of vasomotor symptomatic menopausal women.

To date, how women feel about physical activity as a treatment for their menopausal symptoms remains unknown, since there have been no in-depth qualitative studies exploring their attitudes and experiences about this question. This study aims to explore the feelings, attitudes, and experiences of women who were experiencing vasomotor menopausal symptoms in relation to three key areas of enquiry: (i) using physical activity as a treatment for their HF/NS, (ii) the potential for physical activity to improve their quality of life, and (iii) to consider women’s views about the future implementation of physical activity as a treatment for HF/NS.

## Methods

### Study design and setting

Semi-structured interviews were conducted with participants from the Active Women Study, a randomised controlled trial (RCT) that investigated the effectiveness of two physical activity interventions compared to usual care on HF/NS and other health outcomes in vasomotor symptomatic women [15]. The primary outcome of the trial was the frequency of HF/NS at six-month follow-up. For the trial, 261 women were recruited from 23 general practices across the West Midlands, England. Women were eligible if they were physically inactive at baseline, experiencing at least five HF/NS per day, had not taken MHT in the past 3 months, and were aged between 48 and 57 years. Consenting participants were randomised to one of two 6-month physical activity interventions or a usual care group. Both interventions involved two one-to-one consultations (1 h each) with the study’s physical activity facilitator (who has a MSc in Nutrition, Physical Activity, and Public Health). The consultations focused on equipping women with the skills, knowledge, and confidence needed to participate in regular physical activity in line with public health guidance. The physical activity facilitator informed participants that the definition of moderate intensity physical activity is when there is an increase in their breathing rate, an increase in their heart rate to the level where they can feel their pulse, and an increase in body temperature. Women were encouraged to participate in this type of physical activity 3–5 days per week by the end of the trial period, and were given a pedometer as a motivational feedback tool. In addition to the consultations, one intervention group (physical activity-DVD group) was sent a DVD, a booklet, and five study leaflets at various times throughout the intervention, and the other intervention group (physical activity-social-support group) was invited to take part in three physical activity support groups in their local community. The social-support groups lasted 1–1.5 h and brought women together, allowing them to share experiences about physical activity and the menopause and to offer encouragement to each other. Both interventions provided information about the symptoms of menopause, the importance of physical activity and healthy eating during menopause, and strategies that could be used to motivate participants to maintain a physically active lifestyle. The published protocol and trial report provide more details [15, 16]. Neither of the physical activity interventions resulted in women reporting significantly fewer HF/NS per week compared with usual care at follow-up [15].

### Semi-structured interviews

Individual face-to-face semi-structured interviews were conducted by the first author (AT), who is experienced in conducting research interviews. We aimed to gain a thorough understanding of, and be able to, describe trial participants experiences of physical activity whilst experiencing menopausal symptoms. We therefore used a ‘generic approach’ to reflect this and did not set out to design the research using any specific theoretical perspective [17].

The trial participants were invited by letter to participate in a semi-structured interview once they had completed the six-month follow-up of outcomes in the trial. Participants were purposefully sampled from both physical activity intervention groups and the usual care group, until saturation of the data was achieved. Evidence of data saturation was determined after no new themes emerged from at least two interviews in each of the trial groups [18, 19]. This sampling approach aimed for maximum variation in relation to menopause status, BMI, ethnicity, and socioeconomic status. Twenty-two trial participants were invited for interview. Of these, 17 agreed and were interviewed between November 2011 and February 2013 (Table 1 for participant characteristics). No additional participants were invited for interview once analysis confirmed that data saturation had been achieved. All interviews except one were conducted in participants’ homes. The exception was conducted in a university meeting room at the request of the participant. Measures to neutralise the interviewer’s bias included: conducting the interviews prior to the analysis of the quantitative data from the trial, and the interviewer had never met the participants prior to their interview. Only the interviewer and the participant were present in the room when the interview was conducted. Interviews were semi-structured and a topic guide was used to ensure interview consistency, and that all the core points of interest were discussed. The topic guide was developed based on previous work in this area [20] and based on feedback from our patient advisory group (the full topic guide can be viewed in Additional file 1). Prior to the commencement of the interview, participants were reminded that the purpose of conducting the interviews was to learn about their experiences of being involved in the trial and their views about being physically active during the menopause transition. The interviews lasted 60–90 min. Prompt questions for the interviews were designed to access the feasibility and acceptability of our two physical activity interventions relative to a usual care group. The questions explored four main categories: (i) menopause symptom descriptions and coping mechanisms, (ii) views on physical activity as a treatment for menopausal hot flushes/night sweats, (iii) views and experiences of participating in a menopause and physical activity trial, and (iv) types of physical activity undertaken while being involved in the study. Each interview was digitally recorded, subsequently transcribed verbatim, and inserted in NVivo10 for analysis. Field notes were taken immediately after the interview but were for personal reference only, and therefore not used in the analysis. Participants were not provided with final transcripts for comment or asked to provide feedback on the findings.

**Table 1:** Participant characteristics

	All participants ***n*** = 17	Physical Activity-DVD ***n*** = 6	Physical Activity-Social-support ***n*** = 6	Usual Care ***n*** = 5
**Menopause status**				
Perimenopausal	5	2	1	2
Postmenopausal	12	4	5	3
**BMI**				
Under/normal	6	3	2	1
Over/obese (> 25 kg/m^2^)	11	3	4	4
**Non-white ethnic origin**	5	1	2	2
**IMD quartile**				
1 (least deprived)	3	3	0	0
2	4	0	3	1
3	3	2	1	0
4 (most deprived)	7	1	2	4
**Education**				
Secondary left min age	4	1	1	2
Secondary left after min age	5	1	2	2
University or College of HE	8	4	3	1

### Data analysis

A pragmatic approach to data analysis was taken where the focus was on the key themes that would specifically contribute to the refinement of the intervention and the design of any future trial about physical activity and menopause symptoms. The interview transcripts were read several times to increase familiarity and coded following open-coding procedures [21]. The unit of analysis was the ‘unit of meaning’ also referred as ‘thematic unit’ [22]. Codes that were established initially were applied to subsequent interviews and further codes added as new themes emerged, according to the constant comparative method [23]. Codes relating to one participant were grouped into sub-themes, and sub-themes that related to one concept were grouped into themes. A framework table was built based on the data from the codes within each theme and sub-theme. This framework approach allowed exploration of links between the codes and assisted comparisons of the participants’ views. The coding was conducted by one researcher (AT), with a subset of 10% of the transcripts coded independently by a second researcher (AD) using the developed coding frame. Discrepancies in coding were resolved through discussion and the coding frame adjusted accordingly. Regular meetings were conducted between the researchers to discuss and clarify emerging themes, which were also added to the coding frame. The adjusted coding frame was applied to subsequent interviews and checked for inter-rater reliability by conducting Kappa Analysis on a randomly selected transcript. A Kappa value of 0.93 was achieved. This value, according to Landis and Koch, is an almost perfect inter-rater reliability value [24], indicating agreement in the development of the themes and the codes. Transcripts were coded using NVivo10. This study adheres to the COREQ checklist for reporting qualitative research [25].

## Results

Based on the topics explored during the interviews and the aims of this paper, the key themes of interest identified through the analysis were: (i) thoughts about physical activity as a treatment for menopausal symptoms; (ii) experience of physical activity as a treatment for menopausal symptoms; (iii) the perceived changes to health attributed to physical activity; and (iv) views about implementing physical activity as a treatment for menopausal symptoms. Where women have been quoted, their trial treatment group has also been given (physical activity-DVD group: DVD; physical activity-social-support group: SS; usual care group: UC). Other topics related to trial procedures were explored with women but are not reported here as they are not relevant to the aims of this study.

### Thoughts about physical activity as a treatment for menopausal symptoms

The use of physical activity as a treatment to reduce menopausal symptoms was appealing to participants since they knew of no other ways to manage their symptoms. Participants had tried several commercially available natural remedies without success and felt that MHT was the only available treatment to alleviate their symptoms, but they were adverse to this pharmacological treatment. The majority of participants were bothered by their HF/NS and found them embarrassing. They were keen and felt positive about trying physical activity for several reasons. Although they had no strong beliefs that physical activity could alleviate their symptoms, they were willing to try anything, and they felt that an increase in physical activity could only be positive and could not make their symptoms worse. Participants also preferred to take a natural approach to dealing with their menopausal symptoms, indicating physical activity as a treatment would therefore be appealing:

*a last resort to try (P.3, DVD)*

*another road … I can go down (P.7, DVD)*

*it gives you a feeling of erm, of wellness anyway when you exercise doesn’t it, you do feel better and I can’t see that it could do any harm with menopausal symptoms anyway, it’s only got to be for the better, (P.9, UC)*

*I didn’t want to be turning to drugs myself or taking, I’ve never taken anything like that (P.2, DVD)*

*I want to help myself without taking any medication (P.13, DVD)*

Another possible reason that the women may have been so open to the idea of using physical activity as a treatment is that some believed the study team already knew there was a positive effect:

*I know you don’t pick these ideas up out of the air so you must know there was a good chance that it would help (P.10, SS)*

### Experience of physical activity as a treatment for menopausal symptoms

Most women believed that physical activity reduced their HF/NS, either by reducing the number and/or their intensity. One woman reported an immediate improvement, whereas several others reported experiencing a gradual improvement over time after committing to an increase in daily physical activity levels:

*I think when I’ve done it and I’ve done it constantly and consistently, I think it has made a difference. Because during the exercise I’ve found that sometimes I’d go for a week or 2 weeks without actually getting hot flushes or night sweats. (P.16, SS)*

*I don’t think you realise that it’s making you better at the time. It is something that builds up slowly so it is not something you can do looking for a quick fix. (P.2, DVD)*

However, some women were unsure if their reported reduction in HF/NS could be fully attributed to their increased levels of physical activity; they felt unsure whether it was due to their imagination or other changes in lifestyle:

*I notice when I’ve started the exercising and that, that they’ve started to subside a bit. And I thought, ‘Well is it my imagination or is this in my mind or something?’ but before, I used to have to throw the covers off and, you know, but now I haven’t. (P.3, DVD)*

*Well, the night sweats are much less, without a doubt, and the hot flushes are much less. I mean it’s, it’s difficult for me to say whether it’s that or the dietary changes or a combination of both. Um, but interestingly, I haven’t done much exercise as I said, over the past 4 weeks. And I, they have been worse. And I’ve still been taking the dietary stuff. (P.7, DVD)*

Some women also commented (see quote from P.7, DVD, above) that when their physical activity levels decreased, their HF/NS reappeared.

Many women stated that their night sweats disturbed their sleep resulting in tiredness during the day. Several women went on to comment that they experienced improved quality of sleep after increasing their physical activity levels. It was unclear however from participants’ responses, whether their experience of improved sleep quality was a direct result of a reduction in their NS, or whether their increased physical activity levels led to increased tiredness that then resulted in them sleeping more soundly and consequently they were not then woken by their NS:

*my sleep was being broken because of the hot flushes (P.12, SS)*

*And I think perhaps I sleep more deeply and whether that would have an effect on having less night sweats (P.7, DVD)*

Other health benefits of physical activity as a treatment were also discussed, with most participants talking about sub-themes falling under two categories: physical health benefits and psychological well-being. The perceived physical health benefits from physical activity were in relation to weight or body-shape and fitness or health:

*I found that my legs were coming down... and my butt, I found my butt starting to shrink, as well. And that was the walking (P.12, SS).*

Participants, who commented that physical activity benefited their psychological well-being, felt that it gave them a more positive outlook on life, a higher self-esteem, and elevated mood, made them feel more energised, and provided a stress release:

*I think having these sort of symptoms can draw you down, can make you feel probably a bit depressed, a bit stressful, but I think doing the exercises it lifts you, it lifts your spirits (P.16, SS)*

*I feel good about myself because I’m actually feeling like, as I said to you before, I’m the person before the menopause, (P.3, DVD)*

Although the majority of participants reported improvements in their menopausal symptoms, which they attributed to their increased physical activity levels, a small number of women stated they had experienced no symptom improvements:

*I can’t honestly say hand on heart that the exercise, I have upped what I did, but I can’t honestly say it’s changed anything. (P.5, SS)*

*At first I thought it did but now it doesn’t. (P.13, DVD)*

One woman observed that if she got too hot from exercising, her body could not cool down, which triggered her hot flushes. Despite this, she still felt that increasing her physical activity levels improved her menopausal symptoms when she exercised less vigorously. In contrast to this view, most other participants believed that it was important to undertake vigorous physical activity to achieve a reduction in their HF/NS:

*Now I have cut the exercise down I would say they have helped. When I was going like a bull at a gate I personally thought I made them worse because I seemed to be permanently in a sweat but now I have found the happy medium of what exercise my body can cope with (P.10, DVD)*

*I think you need to increase your heart rate and do cardiovascular sort of exercise for it to actually work. (P.16, SS)*

*I think it’s the intensity whatever you do (P.1, DVD)*

### Perceived changes to health attributed to physical activity

Most participants in the intervention groups commented they had gained menopausal knowledge by participating in the trial and they found this empowering:

*I’d got no idea of course what was doing on, but it was quite good for somebody to sit and talk to us about [the menopause], and talk openly about it and hearing other people talking about what was happening to them. (P.16, SS)*

*most of the information was [in the booklets and on the DVD] and, and I think you’ve covered all that aspect of it really (P.3, DVD)*

*[the booklets and the DVD] were all helpful and they had good information (P.7, DVD)*

In addition, most participants in the physical activity-social-support group commented that they felt more able to cope with their menopausal symptoms after participating in the trial:

*I know how to deal with it now, but previously it would just come on and I wouldn’t know what to do. (P.16, SS)*

*I feel more confident than I did when I first started the study. I understand a lot more. I understand why I am getting the sweats and I can control them better. I know little things to do, how to control them better, so that and I am not making such a fuss of them. Before we used to get in a bit of a flap, which of course didn’t help, erm made them probably last a bit longer, made them a bit more severe than what they was yes. So I am not getting that now because I understand them more. (P.10, SS)*

Notably, this ability to cope was not mentioned by participants in the other physical activity group who received the brochures and DVD, and who were not given the opportunity to meet with other symptomatic women. Although women in the physical activity-social-support group were anxious about attending the group for the first time, most were positive about the sessions, and they appreciated meeting other women experiencing similar issues with their menopausal symptoms. They also welcomed the opportunities these sessions provided to talk openly about their menopausal symptoms and to find out how others were dealing with their symptoms and concerns. These discussions allowed them to realise that they were not alone and that there were other women like them going through similar experiences:

*it was good, just listening to everybody on how other people were doing it, and managing it. (P.12, SS)*

*other people are going through the same problems as yourself, having the same symptoms as yourself, that you weren’t alone out there, and it does happen to other women at your sort of age, so it was good to talk about it openly (P.16, SS)*

*so we were three completely different people, suffering exactly the same way and I thought ‘oh it is alright I am not standing out in the crowd sort of thing’. … it was quite relieving actually because I did feel you know ‘is this normal?’ (P.10, SS)*

After attending the support groups, many of the women also reported changes to how they perceived the severity of their symptoms:

*I’m aware through doing the study, some people have really suffered, much worse than I have, so I feel now I’m sort of floating through it compared to some people (P.5, SS)*

*you have got nothing to compare it to where at least with the study you could compare it (P.10, SS)*

As a result of participating in the trial, most women reported changes to their physical activity levels that were achieved by an increased awareness of identifying opportunities to incorporate physical activity into their lives and/ or a greater understanding of what aerobic walking is:

*making me think again about what I could do to make, because you always think ‘oh I’m doing enough’ but it’s just making you think about what more you could do (P.5, SS)*

*more aware of the sort of walking that you are doing which I have never thought about before, walking is just walking. Erm, but now I am … aware of whether you are walking properly or whether you are dawdling. (P.2, DVD)*

Participants felt many menopausal women have difficulty identifying or rating the intensity of vigorous physical activity and that they may believe they are doing more physical activity than is actually the case. This disconnect was attributed to the elevated body temperature generally experienced by menopausal women, or alternatively the tendency of some to have been on their feet all day:

*some women … … they think by working in the house and cleaning, they’re doing exercise. That’s not … it’s not right. So they should go really proper exercise (P.14, UC)*

*you do tend to get a lot warmer within maybe a lot quicker than people who aren’t got hot sweats, so I probably think that you are working dreadfully hard because it is just pumping out of you and you are not really. (P.10, SS)*

### Views about implementing physical activity as a treatment for menopausal symptoms

The participants made numerous positive comments about the way in which the study had implemented the physical activity interventions and supported increases in physical activity levels. Participants reported that they liked that the physical activity interventions were tailored to their individual needs and lifestyles and were based around activities they already did or were interested in doing:

*it was quite nice that it was based around what I did already and what I was interested in already and setting targets for myself, which you sort of get somehow determined to achieve (P.5, SS)*

Additionally, women valued that the support was facilitative, rather than intrusive or pressured, and that more support was available via phone or email.

Participants commented that they would prefer physical activity to be the first treatment suggested by GPs, rather than the GPs first suggesting that medication should be used to control their symptoms:

*I personally would like that as the, as his first suggestion, I like that idea. If he suggests you diet, exercise as a, instead of saying yeah here’s a tablet … … he says look try this for a couple of months, see how that goes and come back to me. I think, I’d like that as a, you know like a gradual step if I had to go to [MHT] … but not go straight there. (P.6; UC)*

Two women from the physical activity-DVD group specifically suggested that this type of physical activity intervention should be implemented by healthcare professionals to encourage increased levels of physical activity as part of the ‘well women programme’. Both women believed that it was important that the programme include the support of a physical activity facilitator, who would help women to increase their physical activity levels:

*I think it’s a very good time, you know we have all these well woman things don’t we and mammograms and hideous things and you can actually do with doing this (P.1, DVD).*

*I would say it should be available at the surgeries as well. You should have somebody who’s not a doctor or nurse, … … … this needs to be sort of put in to more surgeries and telling people that, ‘We’re here’. ‘We’ll get you started’. (P.3, DVD)*

## Discussion

The current study aimed to explore the feelings, attitudes, and experiences of women with vasomotor menopausal symptoms about using physical activity as a treatment for their HF/NS. This study also aimed to investigate their beliefs about whether physical activity could alter their quality of life, and their thoughts about future implementation of physical activity as a treatment for HF/NS. Most women believed that participating in physical activity reduced their HF/NS. Whilst a few women did not feel that participating in physical activity reduced their HF/NS, all participants generally expressed positive views about the role of physical activity as a treatment for their HF/NS. Participants held the view that participation in physical activity improved their overall health and well-being and were supportive of physical activity being used as treatment for vasomotor menopausal symptoms by healthcare professionals, rather than medication.

### Thoughts about physical activity as a treatment for menopausal symptoms

Participants were positive and open about the idea of healthcare professionals recommending, and then using physical activity, as a treatment for managing their menopausal symptoms. Participants were frustrated that they had tried several commercial natural remedies without impact on their symptoms and felt there were no other treatment options besides MHT. However, there were strong feelings amongst participants of not wanting to use MHT and they felt resigned that they would have to continue to experience HF/NS. For these reasons they were willing to try any alternative treatment. Participants were open to the suggestion of using physical activity because it was considered a natural or non-pharmacological approach and there was ‘nothing to lose by trying it’. In other words, the potential benefits of participation in physical activity outweighed any possible risks, which were viewed as substantially less than the MHT. In line with the results from this qualitative study, other studies have found that many women preferred to manage their menopausal symptoms naturally [26–28]. The belief held by the women that they had ‘nothing to lose’ is perhaps a consequence of the accepted benefits associated with physical activity [12–14] and participation would nevertheless improve their overall health even if it did not reduce the HF/NS.

Other than MHT, women did not refer to any other types of non-hormonal pharmacological treatments, suggesting they believed MHT was the only medication available to them. Whilst MHT is acknowledged as the first choice of treatment of menopausal symptoms for women with no contraindications [29, 30], other pharmacological treatments have been identified as effective alternatives, for example, selective serotonin reuptake inhibitors and serotonin-norepinephrine reuptake inhibitors [29]. This apparent lack of knowledge about other effective treatments for HF/NS highlights the importance for healthcare professionals to ensure their patients have sufficient knowledge about all the possible treatments that may be available.

### Experience of physical activity as a treatment for menopausal symptoms

Most of the women interviewed in the physical activity groups felt that implementing physical activity as a treatment, and thereby increasing their physical activity levels, reduced their menopausal symptoms. Specifically, participants reported that they experienced less and/or lower intensity HF/NS. Whilst a Cochrane review [11] reported there was insufficient evidence to conclude physical activity was effective in reducing HF/NS, several RCTs [31–34] have highlighted a reduction in HF/NS consistent with the experiences reported by women in this study. A small 12-week RCT (*n* = 75) found the average number of HF decreased by about a third of baseline levels in ten previously sedentary women who participated in physical activity three-times per week [33]. A larger four-month exercise RCT (*n* = 164) also reported reduced vasomotor symptoms in women allocated to the walking and yoga groups, but there was no corresponding change in the control group [31]. Likewise, a six-month RCT (*n* = 176) where women participated in an aerobic training intervention reported a significantly larger decrease in NS for participants than for the control group as recorded in the participants’ mobile phone diaries [32, 34].

However, other RCTs, including the one on which this qualitative study was embedded, found that physical activity interventions lasting between 3 and 6 months did not result in significant differences between the groups in the frequency of HF/NS at follow-up [15, 35, 36]. Although the RCT in which this qualitative study was embedded found no significant differences, there was evidence of a non-significant reduction of HF/NS frequency when compared to usual care; by 22% for the physical activity-DVD group, and by 13% for the physical activity-social-support group [15] but the trial was powered to detect a higher 50% reduction in HF/NS. Nevertheless, it does appear from the findings of this qualitative study that at least some women perceived they experienced a meaningful reduction of HF/NS frequency that was less than 50%.

The difference between the observations this study reports regarding physical activity and the impact on HF/NS with the overall findings from the RCT, in which this study was embedded, may have other explanations. These explanations include the suggested associations between reduced menopausal symptoms, both with improvements in physical fitness [31, 32, 37] and with improvements of thermoregulatory function [38, 39]. Improved physical fitness was associated with less severe physiological HF symptoms [39], while a greater control of thermoregulatory function coincided with reduced HF frequency and severity [38] in symptomatic postmenopausal women (*n* = 21) participating in a 16-week aerobic training controlled trial [38, 39]. There were no measurements of physical fitness, physiological symptoms of HF/NS, or thermoregulatory function in the RCT in which this study was embedded. Hence, it is unknown if changes of these parameters occurred in women recruited to the physical activity interventions of this RCT. It is possible that by chance the majority of women interviewed from the physical activity intervention groups did experience improvements in these parameters, and hence the associated reductions in menopausal symptoms. This association between reduced menopausal symptoms and improvements in physical fitness [38] may also explain why some women in this study observed their HF/NS reoccurred when they decreased their physical activity levels. This reoccurrence of symptoms also occurs on the discontinuation of MHT [40].

In addition to improvements to HF/NS, the women in the intervention groups described improvements in their general symptoms of the menopause such as insomnia, mood, and weight gain after they increased their physical activity levels. The experiences from participating in physical activity reported by women in this study are consistent with public-health guidance that states regular participation in physical activity has numerous physical, social, and psychological benefits to health [12, 13, 32, 36]. Other studies have also reported increases in physical activity levels improves sleep quality in both non-menopausal [41] and menopausal women [35, 36, 42, 43], as well as other general or non-specific symptoms attributed to the menopause, such as weight gain, headache, depressive mood, and nervousness [32, 35, 44–46]. Therefore, regardless of whether increasing physical activity levels quantitatively reduces the number and intensity of HF/NS, this study highlights the potential that physical activity interventions could improve women’s perceptions of their HF/NS symptoms and other menopausal symptoms. Even the perception that bothersome menopausal symptoms have reduced may contribute to overall improvements in symptoms in menopausal women, as expressed by participants in this study.

### Perceived changes to health attributed to physical activity

Participants reported that they felt participation in the trial increased their knowledge of the menopause and this was felt by them to be empowering because they learnt more about what was changing inside their body, what to expect as a result of the menopause transition, and consequently how to deal with these changes to their body [47]. It may be that this increased knowledge about the menopause meant that women felt they had contributed to their ability to cope better with their symptoms, a finding that has been reported by other studies [26, 45, 48]. Participants who attended the intervention that included the physical activity support groups commented that these groups contributed to improvements in their ability to cope with their menopausal symptoms. Other studies have reported that attending similar support groups was beneficial in improving menopausal knowledge, giving women a more positive attitude towards menopause, reducing stress [48–50], and reducing hot flushes [51]. By socialising at the support groups with other women experiencing similar menopausal symptoms, participants were able to normalise their symptoms, often resulting in a change of how they perceived the severity of their symptoms. The findings of this study supports the provision of support for women, to help them follow an active and healthy lifestyle during the menopause transition.

As well as understanding more about the menopause, participants reported that they had learnt more about what moderate and vigorous intensity physical activity ‘felt like’. Not only did participants not understand the different levels or types of intensities of physical activities such as of walking, they also believed that many menopausal women overestimate the intensity of their physical activity because their bodies feel warmer all the time and they are more prone to sweating. This potential overestimation of physical activity by menopausal women may have consequences in studies that only subjectively measure intensity of physical activity in participants. Future research should consider the impact that this discrepancy might have on menopausal women’s experiencing and understanding of different types of physical activities.

### Views about implementing physical activity as a treatment for menopausal symptoms

This study found that menopausal women would like healthcare professionals to encourage women to engage in physical activity as a first treatment option for their menopausal symptoms because of the wide range of health benefits that physical activity can provide, before offering medication such as MHT. Furthermore, participants in the physical activity-DVD intervention group commented that their intervention should be included within the NHS Health Check programme offered to women over the age of 45 years in England. Participants felt that such a programme should include patient-centred consultations with a healthcare professional that provide emotional support to women approaching menopause, information about healthy behaviours, their changing bodies and subsequent symptoms, and specific advice about how to increase their physical activity levels. Participants felt the consultations should be accessible, with the option of support groups similar to those offered in this study, and include follow-up appointments to encourage and support long term behavioural changes in physical activity [52]. Educational support groups have been associated with improved menopausal attitude and coping with physical and psychosocial aspects [50], and reduced hot flushes [51]. Furthermore, a 12-week health promotion programme noted that women who received four healthcare support consultations in addition to written information on a range of health topics (that included regular exercise, healthy eating and the menopause), reported larger reductions in menopausal symptoms from baseline than the women who only had access to the written information [53].

To ensure the best practice of using physical activity as a treatment option for menopausal symptoms, it will be important to educate healthcare professionals about the importance of tailored information and support that includes planned follow-up appointments rather than only providing the women generic written information about healthy lifestyle behaviours and the menopause. It also does need to be highlighted that although MHT is documented as the most effective treatment [30] and there is a lack of conclusive evidence about the effectiveness of physical activity for reducing HF/NS [11], there is strong evidence for physical activity reducing other menopausal symptoms, for example insomnia, weight loss, depression and cardiovascular disease [32, 35, 36, 42–46].

### Strengths and limitations

A strength of this study is that it is the first to directly explore the attitudes and preferences of symptomatic menopausal women about prospective treatments for their menopausal symptoms, with a focus on understanding the role of physical activity. To enhance credibility of the findings, the interviews took place in natural settings, at a time and place determined by the participant. The data were iteratively collected allowing us to systematically identify similarities and differences in participants’ responses. The participants invited to take part in this study were selected based on a range of socio-demographic factors, including menopause status, BMI, ethnicity and socioeconomic status, and without knowledge of the quantitative findings from the trial. By attempting to select women from a range of sociodemographic backgrounds, the opportunity for wide a range of opinions and perspectives to be conveyed has been created. Participants from all three trial groups, including those randomised to receive usual care were included in the views expressed here. This is important because those who received the intervention may express more favourable views that those who did not.

This study also has some limitations that should be considered when interpreting the findings. Participants were enrolled in a trial that assessed the effectiveness of physical activity as a treatment for HF/NS. This prior interest in using physical activity as a treatment option may have influenced participants’ responses, such that, for example, the participants may have been more willing to partake in physical activity than the general population. It therefore may be worthwhile to further explore the topics raised in this study from a wider population of menopausal women.

## Conclusion

In this study menopausal women reported that they valued being encouraged and supported to participate in regular physical activity on the basis that physical activity may reduce their menopausal symptoms and provide other health benefits. Symptomatic menopausal women would also welcome healthcare professionals offering more information about the menopause transition. Participants stated they would prefer healthcare professionals to suggest physical activity as a first option to treat menopausal symptoms.

## Supplementary information

**Additional file 1.** Interview Topic Guide.

## Data Availability

The datasets used and/or analysed during the current study are available from the corresponding author on reasonable request.

## Abbreviations

BMI: Body mass index
DVD: Physical activity-DVD group
HF: Hot flushes
IMD: Index of multiple deprivation
MHT: Menopausal hormone therapy
NS: Night sweats
RCT: Randomised controlled trial
SS: Physical activity-social-support group
UC: Usual care group
