# Supplementary material for: Women’s views about physical activity as a treatment for vasomotor menopausal symptoms: a qualitative study
Source: BMC Womens Health. 2020 Sep 14;20:203. doi: 10.1186/s12905-020-01063-w (PMC7488995; doi:10.1186/s12905-020-01063-w)
Supplement: Supplementary file 1 — Additional file 1. Interview Topic Guide. [file 12905_2020_1063_MOESM1_ESM.docx]

# Additional File 1: Interview Topic Guide

## Initial questions to warm up

What menopausal symptoms have you been experiencing?

How long?

How much do/did they bother you?

## Questions about menopause diagnosis

Where you going to the doctors about your symptoms? Still going?

Did you have any other forms of support to help deal with menopause symptoms? (family, friends, clinics, groups)

Did friends and family know you were having lots of HFs / NS?

## Questions about the menopause trial

How did you feel about being asked to take part in a trial about menopause symptoms?

Why did you decide to take part?

What did you understand the aims of the study to be? Is this still your understanding?

What were you hoping to achieve by taking part? e.g. improved well being, reduced hot flushes / night sweats (HF/NS)

What exercise, if any, were you doing at the time?

In what ways did you think exercise might help your symptoms?

Did you think it might help in other ways? e.g. meet people, improved physical exercise.

Do you enjoy exercising?

## Questions about views on exercise as a treatment for menopausal hot flushes / night sweats

When you were 1^st^ invited, what did you think about exercise being used as a treatment for menopausal hot flushes / night sweats – did you think it might work? Why/why not?

Now that you’ve completed the study, how have your views changed?

Do you see it as having a particular appeal to certain groups of women? e.g. active people, people working part-time?

Did you find only certain types of exercises made symptoms better / worse? If so, which activities?

Do you think the intensity or length of the exercise made a difference to HFs / NS? If so, in what way?

What do you see as the pros and cons of exercise as a treatment for hot flushes / night sweats? e.g. time intense Vs ‘healthy’ / health benefits.

Have you ever used exercise as a way to improve your health? Why/why not? In what way? (lose weight, how you feel)

What about your how you feel in general? In what way? ? (depression / to get out and about)

Do you think suffering from HF/NS can impact on someone’s ability to engage with exercise? Why/why not?

## Questions about experiences of the trial – wearing the Actiheart device

How did you find wearing the actiheart?

Did you see the data that the actiheart produced? Could you understand what it meant?

Did wearing the actiheart change your behaviour in any way? In what way?

## Questions about experiences of the trial

How did you feel when you were allocated to the usual care group / an exercise group?

Which group were you in? Was this the group you wanted? Why/why not?

Questions to participants in physical activity groups only:

What did you think would happen at the 1^st^ appointment with the physical activity facilitator?

What did you think the physical activity facilitator’s role / purpose was?

What were your expectations about what information and support would be given?

Tell me about the visits? What did you do, what information was given, what do you think the physical activity facilitator was trying to do, what goals were set etc?

What was helpful and unhelpful about these visits?

Questions to participants in physical activity-DVD group:

What did you think about receiving the brochures and DVD in the post? (motivating / nuisance)

When you received items in the post from us, how did they make you feel? (happy / nuisance / more junk mail)

Did they encourage you to do more exercise? In what way?

About the content of the brochures and DVD, what do you think could have been improved?

Did you feel that the brochure & DVD were repetitive?

Overall, what could be done differently?

What else should be offered?

Questions to participants in physical activity-social support group:

How did you feel about attending the support groups?

How many did you attend?

Did you think that the support groups were well lead, organised?

Which discussion topic(s) did you find the most useful?

What other topics would you have liked to discuss at the support groups?

How helpful did you find the support groups?

Overall, what could be done differently?

What else should be offered?

## Questions about the exercise undertaken and barriers and support

Questions to participants in the usual care group only:

Did you exercise after joining The Active Women Study? If so:

- - - Is this more than you were doing before?

Questions to all participants (physical activity and usual care groups):

Since joining The Active Women Study, can you tell me what exercise you have engaged in? Why these activities, how regularly do you do them?

Do you look forward to exercising or do you dread it

How does exercise make you feel? - During / after

How do you think exercise has affected your hot flushes / night sweats?

Have your symptoms got better / worse? In what way? E.g. coping better, intensity of sweats.

If a range of activities performed:

- - - Which activities did you enjoy the most?
    - Which activities did you feel had the most impact on your mental health? Why?
    - Where there any you did not enjoy? Why?
    - Where they any you felt had a negative impact on your wellbeing? Why?

What makes it difficult for you to exercise? (feeling tired, too busy, children)

Are there issues that affect you engaging in physical activity? e.g. cost, facilities, time, feeling safe, feeling low?

What helps/motivates you to engage in exercise? e.g. feeling good afterwards, friend’s encouragement?

Have you told family/friends that you are taking part in The Active Women Study? If so, what was their reaction? Have they encouraged/discouraged you in any way?

## Final Questions

How are you feeling now? Why do you think this is?

Questions to participants in the usual care group only:

During the study have your symptoms got better / worse? In what way? E.g. coping better.

Do you plan to do any / more exercise in the future? What form?

Questions to participants in physical activity groups:

What exercise do you hope to do/maintain from now on?

What factors do you think will help you maintain these changes to your exercise habits? (i.e. continuing to feel good, hot flushes staying reduced, sleeping better)

- - - What will prevent you from continuing with your new exercise habits?

Would you recommend physical activity to other women suffering with hot flushes/night sweats? What advice would you give to them?

Questions to all participants:

Overall thinking about the study -what do you think we could improve on?

Is there anything we did particularly well?

Is there anything else you would like to say about menopausal hot flushes / night sweats, your experiences of a particular treatment, views on treatments available and/or the trial?
